# C‐reactive protein/albumin ratio as a novel predictor for nutritional status of geriatric patients

**DOI:** 10.1002/brb3.70017

**Published:** 2024-09-11

**Authors:** Tezcan Kaya, Sena Boncuk Ulaş, Ahmet Nalbant, İlhan Yıldırım, Kubilay İşsever, Cengiz Karacaer, Cahit Bilgin, Aslı Vatan, Türkan Acar, Bilgehan Atılgan Acar, Yeşim Güzey Aras, Mehmet Köroğlu

**Affiliations:** ^1^ Department of Internal Medicine Sakarya University Faculty of Medicine Sakarya Turkey; ^2^ Department of Neurology Keşan State Hospital Edirne Turkey; ^3^ Department of Internal Medicine Görele Dr. Ergun Özdemir State Hospital Giresun Turkey; ^4^ Department of Internal Medicine Giresun University Faculty of Medicine Giresun Turkey; ^5^ Department of Internal Medicine Sakarya Training and Research Hospital Sakarya Turkey; ^6^ Department of Chest Diseases Sakarya University Faculty of Medicine Sakarya Turkey; ^7^ Department of Infectious Disease Sakarya University Faculty of Medicine Sakarya Turkey; ^8^ Department of Neurology Sakarya University Faculty of Medicine Sakarya Turkey; ^9^ Department of Microbiology Sakarya University Faculty of Medicine Sakarya Turkey

**Keywords:** Aged people, albumin, geriatric assessment, malnutrition, nutrition assessment

## Abstract

**Background and aims:**

The C‐reactive protein to albumin ratio (CAR) is a novel parameter that has been reported as a significant prognostic marker in some diseases. The purpose of the present research was to investigate the predictive value of this ratio with regard to nutritional status in geriatric patients.

**Methods and results:**

A total of 154 geriatric patients (age ≥65 years) who consecutively presented to the internal medicine outpatient clinic were included in this cross‐sectional study. The Mini Nutritional Assessment (MNA) was used as a reference to determine the nutritional status of the patients. Based on the MNA results, the patients were divided into two groups: normal nutrition and malnourished or at risk of malnutrition. The median CAR of malnourished patients or those at risk of malnutrition was significantly higher than that of patients with normal nutritional status (*p* = .012). A significant negative correlation was also observed between the MNA score and the CAR (*r* = −0.196, *p* = .015). The receiver operating characteristic curve analysis indicated that the CAR was a significant predictor of malnourishment or the risk of malnutrition (*p* = .012).

**Conclusion:**

The CAR could predict which geriatric patients were malnourished or at risk of malnutrition. CAR may be used as a new tool in the nutritional screening of geriatric patients.

## Graphical abstract

The median C‐reactive protein/albumin ratio (CAR) of malnourished patients or those at risk of malnutrition was significantly higher than that of patients with normal nutritional status (p=0.012). The CAR could predict which geriatric patients were malnourished or at risk of malnutrition.

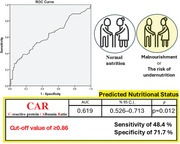

## 1 INTRODUCTION

Malnutrition in geriatric individuals has been linked to various adverse outcomes (Dong et al., 2021; Kramer et al., 2022; Lim et al., 2012; Sharma et al., 2017). For instance, hospitalized patients experiencing malnutrition tend to face increased costs, prolonged lengths of stay, and elevated rates of readmission and mortality (Lim et al., 2012; Sharma et al., 2017). Poor nutritional status is associated with diminished physical performance and serves as a predictor of all‐cause mortality (Dong et al., 2021; Kramer et al., 2022). In light of these detrimental consequences, it is imperative to assess the nutritional status of individuals accurately. The literature suggests several methods for ensuring precise nutritional screening of geriatric individuals (Bellanti et al., 2022; Cederholm et al., 2017). Multiple validated screening tools are available and commonly employed (Bellanti et al., 2020; Rubenstein et al., 2001; Stratton et al., 2004; Vellas et al., 1999). However, a universally accepted gold standard method for nutritional assessment is lacking, leading to varied recommendations in the existing literature (Cederholm et al., 2017; Cederholm et al., 2019).

The serum albumin level is frequently utilized as a biomarker to assess the nutritional status of patients (Bellanti et al., 2022; Cederholm et al., 2017; Cederholm et al., 2019). It is a negative acute phase reactant, and its level decreases mainly in chronic inflammation and malnutrition. In this context, C‐reactive protein (CRP) serves as a sensitive marker indicative of body inflammation and also is a well‐known acute phase reactant and has been associated with various diseases (Eckart et al., 2020; Hizli et al., 2021). The CRP‐to‐albumin ratio (CAR), a novel parameter that has garnered attention in recent years, has been subject to investigations (Liao et al., 2021; Luan et al., 2021; Yu et al., 2021). Studies have reported the CAR alone as a significant prognostic biomarker for poor outcomes and overall survival in specific diseases (Liao et al., 2021; Luan et al., 2021; Yu et al., 2021). CAR has been suggested to be a more reliable risk indicator for inflammatory conditions than CRP or albumin alone. It also appears as a combination of systemic inflammation and nutritional status and has been associated with clinical outcome in many diseases (Haider Kazmi et al., 2022; Kunutsor & Laukkanen, 2022).

Malnutrition in the geriatric population is a serious health problem, and it is important to assess nutritional status in this population as it can lead to various diseases. Dysphagia also has a major impact on nutritional status and malnutrition, and the geriatric population is also at risk in this respect. Therefore, the detection of malnutrition in the geriatric population is of great importance (Bayram et al., 2021; Epçaçan et al., 2023). Given these findings, we hypothesize that the CAR could serve as a valuable indicator for nutritional screening. Hence, this research aims to explore the potential of the CAR in predicting the nutritional status of geriatric patients.

## 2 MATERIALS AND METHODS

This cross‐sectional study encompassed geriatric patients (aged ≥65 years) consecutively admitted to the internal medicine outpatient clinic of a training and research hospital between May 2022 and August 2022. Exclusion criteria comprised individuals with acute/chronic infection, malignancy, rheumatic diseases, steroid use, chronic hematological diseases, hospitalization within the last month, liver cirrhosis, protein‐losing enteropathy, nephrotic syndrome, chronic kidney failure, and CRP values exceeding 10 mg/L. Recorded data included the patients' demographic and clinical characteristics, nutritional status, and laboratory information. Approval for conducting the study was obtained from the local university's Ethics Committee of the Faculty of Medicine (approval No. E‐71522473‐050.01.04‐128291). Participants were adequately informed about the study, and their participation was contingent upon providing signed informed consent.

The Mini Nutritional Assessment (MNA) served as the tool for assessing the nutritional status of the patients (Vellas et al., 1999). Comprising 18 items related to anamnesis, dietary characteristics, and anthropometric measurements, including mid‐arm circumference, body mass index (BMI), calf circumference, triceps skin fold (TSF), and the MNA covered various aspects, including number of meals, daily menu content, fluid intake, fruit and vegetable consumption, presence of neuropsychological conditions and other diseases, mobility, and ability to eat independently (Vellas et al., 1999). The validity and reliability of MNA have been demonstrated in previous studies (Ekici et al., 2021). Additionally, there are studies evaluating its validity in the geriatric population (Sarikaya et al., 2015). Total MNA scores falling within the range of 24−30 indicated normal nutritional status, scores between 17−23.5 signaled the risk of malnutrition, and scores below 17 signified malnutrition (Vellas et al., 1999). Two trained researchers administered the MNA questionnaire and conducted measurements for height, weight, BMI, mid‐arm circumference, TSF, and calf circumference. BMI was calculated by dividing weight (kilograms) by the square of the height (meters). The International Biological Program was analyzed for the evaluation of anthropometric measurements (Weiner & Lourie, 1969). Subsequently, patients were categorized into two groups: those with normal nutrition (MNA scores of 24−30) and those classified as malnourished or at risk of malnutrition (MNA scores ≤23.5). The CAR and other parameters were then compared between the two groups.

The results of patients' laboratory examinations were obtained from the hospital automation system, with those lacking measurements for serum albumin and CRP values being excluded from the study. Serum albumin, creatinine, and total cholesterol were assessed using a spectrophotometric technique. The CRP level was determined using an immune‐turbidimetric method on an Olympus AU5800 auto‐analyzer. Accepted reference ranges were 3.2−4.6 g/dL for albumin and 0−5 mg/L for CRP. The CAR was introduced as a novel parameter, calculated by dividing the CRP value by the serum albumin value. The study focused on exploring the relationship between the CAR and nutritional parameters, as well as evaluating the CAR's efficacy in predicting the nutritional status of the included patients.

Continuous variables were presented as the mean ± standard deviation or median (25th−75th percentile), while categorical variables were expressed as frequency and percentage. The normality of variable distributions was assessed using the Kolmogorov–Smirnov test. Chi‐square tests were employed for categorical variables, and the Mann–Whitney *U* test or Student's *t*‐test was utilized for continuous variables. Spearman's correlation test was applied to evaluate the association between the CAR and MNA scores. The receiver operating characteristic (ROC) curve was employed to identify the optimal cut‐off value for the CAR in predicting nutritional status. To assess the CAR's performance regarding the nutritional status of patients, ROC curves, sensitivity, and specificity values were calculated. Binary logistic regression analysis was conducted to determine the association between the CAR and patients' nutritional status. A significance level of *p* < .05 was applied to all statistical analyses, which were carried out using the Statistical Package for Social Science (SPSS version 22).

## 3 RESULTS

The study consisted of 154 patients who met all criteria. The patients’ mean age was 73.2 ± 5.9 years, and 99 (64.3%) were female. The clinical features of the participants are shown in Table 1. Among the participants, 40.2% (*n* = 62) were malnourished or at risk of malnutrition. The mean age of the malnourished or at‐risk‐of‐malnutrition patients was higher than that of patients with normal nutrition (75.2 ± 6.3 vs. 71.9 ± 5.3 years, *p* = .001) (Table 1). No intergroup differences were found in terms of patient sex or other demographic characteristics. The CAR and nutritional parameters of the patients based on the MNA results are shown in Table 2. The median CAR value of the participants who were malnourished or at risk of malnutrition was significantly higher than that of participants with normal nutritional status (0.84 [0.74−2.22] vs. 0.77 [0.70−0.90], *p* = .012) (Table 2).

**TABLE 1:** Clinical characteristics of patients according to nutritional status.

Characteristic	Total (*n* = 154)	Well nourished (MNA score 24–30) (*n* = 92)	Malnourished or at risk of malnutrition (MNA score ≤23.5) (*n* = 62)	*p*‐value
Gender (male/female)	55/99	35/57	20/42	.573
Age (years)	73.2 ± 5.9	71.9 ± 5.3	75.2 ± 6.3	.001
Hypertension *n* (%)	100 (64.9)	58 (63)	42 (67.7)	.669
Diabetes *n* (%)	57 (37)	33 (35.9)	24 (38.7)	.851
CVD *n* (%)	17 (11)	7 (7.6)	10 (16.1)	.164
CVA *n* (%)	11 (7.1)	6 (6.5)	5 (8.1)	.439
White blood cells (K/μL)	6.79 ± 1.77	6.82 ± 1.71	6.75 ± 1.88	.596
Hemoglobin (g/dL)	13 ± 1.3	13.2 ± 1.1	12.8 ± 1.5	.054
Platelets (K/μL)	250 ± 59	250 ± 59	250 ± 60	.938
AST (U/L)	20.2 ± 9.1	20.1 ± 9.2	20.2 ± 9	.889
ALT (U/L)	14 (11–21)	14.5 (12–22)	13 (10–21)	.197
Creatinine (mg/dL)	0.8 ± 0.2	0.8 ± 0.2	0.9 ± 0.2	.068

*Note*: Data were shown as mean ± SD, number (percentage), or median (percentiles 25–75).

Abbreviations: ALT, alanine amino‐transferase; AST, aspartate aminotransferase; CVA, cerebrovascular accident; CVD, cardiovascular disease; MNA, Mini Nutritional Assessment.

**TABLE 2:** C‐reactive protein to albumin ratio and nutritional parameters of 154 geriatric patients according to nutritional status.

Parameters	Well nourished (MNA score 24–30) (*n* = 92)	Malnourished or at risk of malnutrition (MNA score ≤ 23.5) (*n* = 62)	*p*‐value
MNA score	26 ± 1.4	21.1 ± 2	<.001
CAR	0.77 (0.7–0.9)	0.84 (0.74–2.22)	.012
CRP (mg/L)	3.2 (3.2–3.6)	3.2 (3.2–3.5)	.818
Albumin (g/dL)	4.2 ± 0.3	4 ± 0.4	.037
Neutrophil count (K/uL)	3.85 ± 1.48	3.93 ± 1.36	.715
Lymphocyte count (K/uL)	2.22 (1.73–2.59)	1.86 (1.26–2.58)	.051
Total cholesterol (mg/dL)	218 ± 39	211 ± 48	.264
LDL‐C (mg/dL)	134 ± 33	131 ± 35	.401
Triglyceride (mg/dL)	133 (98–179)	122 (88–171)	.306
Weight (kg)	76.6 ± 11.7	65.2 ± 13.1	<.001
Height (m)	158 ± 9	155 ± 8.7	.014
BMI (kg/m^2^)	30.5 ± 4.6	27.2 ± 5.3	<.001
MAC (cm)	30.5 ± 3	27.8 ± 3.7	<.001
Calf circumference (cm)	36.8 ± 3.3	34.4 ± 4.3	<.001
TSF (mm)	18 (11.2–23.8)	12 (8.5–19.5)	.005

*Note*: Data were shown as mean ± SD or median (percentiles 25–75).

Abbreviations: BMI, body mass index; CAR, C‐reactive protein to albumin ratio; CRP, C‐reactive protein; LDL, low‐density lipoprotein; MAC, mid‐arm circumference; MNA, Mini Nutritional Assessment; TSF, triceps skin fold.

**FIGURE 1:**
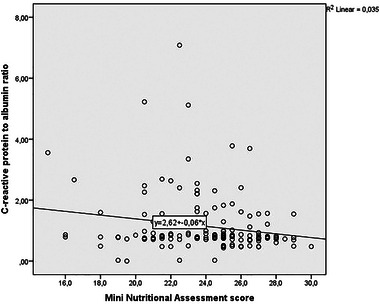
Correlation graph of C‐reactive protein to albumin ratio and Mini Nutritional Assessment score (*r* = −0.196, *p* = .015).

**FIGURE 2:**
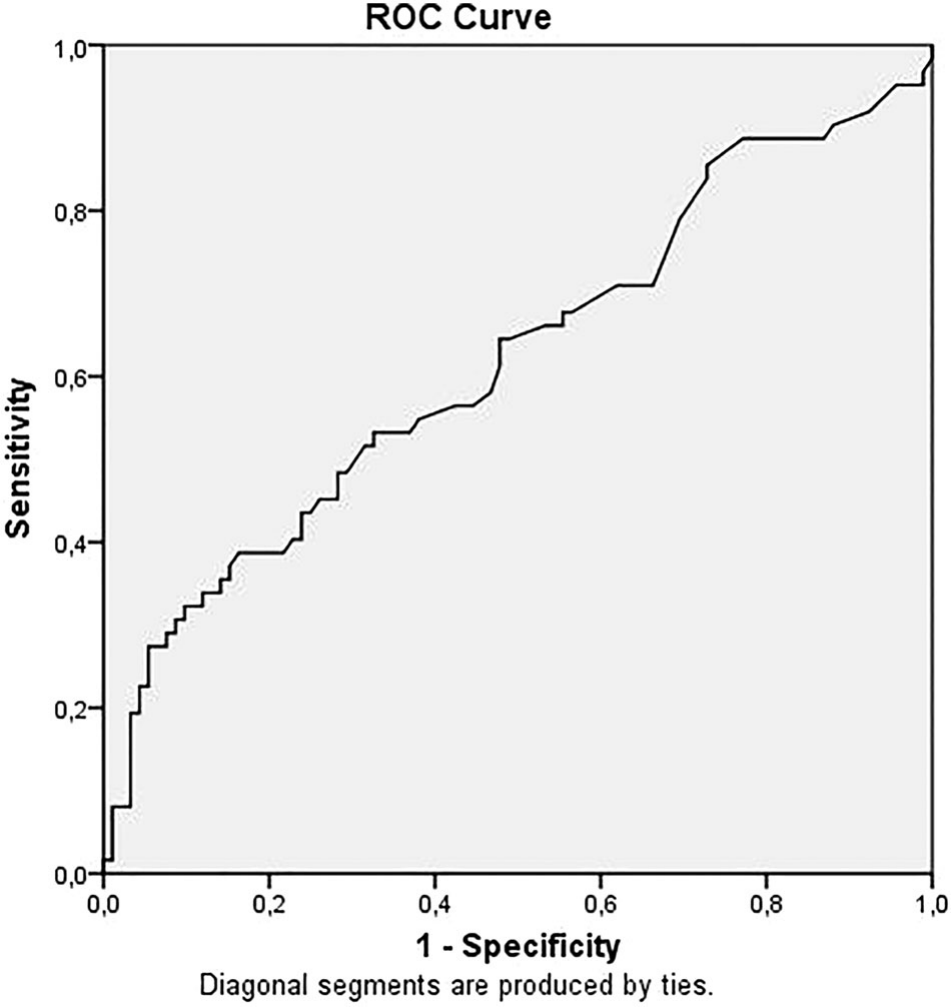
Receiver operating characteristics curve of C‐reactive protein to albumin ratio for predicting nutritional status in geriatric patients (AUC = 0.619, *p* = .012). ROC, receiver operating characteristic.

There was a significant correlation between the MNA results and the CAR (*r* = −0.196, *p* = .015) (Figure 1). The ROC curve analysis revealed that the CAR could be considered a significant predictor of malnourishment or the risk of undernutrition (area under the curve = 0.619; *p* = .012; 95% confidence interval, 0.526−0.713) (Figure 2). A CAR optimum cut‐off value of ≥0.86 predicted nutritional status with a sensitivity of 48.4% and specificity of 71.7%. Moreover, binary logistic regression analysis suggested that the CAR alone was an independent predictor of undernutrition or the risk of malnutrition (odds ratio, 0.714; 95% confidence interval, 0.532−0.958; *p* = .025).

## 4 DISCUSSION

The current study uncovered a significant elevation in the CAR among geriatric patients who were either malnourished or at risk of malnutrition compared to those with a normal nutritional status. The CAR emerged as a biomarker capable of independently predicting malnourishment or the risk of malnutrition in these patients. Moreover, the CAR was identified as an independent predictor of malnourishment or the risk of malnutrition in the geriatric population. Consequently, the CAR may offer a novel parameter for stand‐alone use in the nutritional screening of the geriatric population.

The well‐established association between malnutrition in geriatric individuals and various adverse clinical outcomes underscores the importance of nutritional screening and appropriate support for this population (Dong et al., 2021; Kramer et al., 2022; Lim et al., 2012; Sharma et al., 2017). Currently, specific validated tools are utilized to assess the nutritional status of geriatric individuals (Bellanti et al., 2020; Rubenstein et al., 2001; Stratton et al., 2004; Vellas et al., 1999). However, a universally accepted method for this purpose is lacking. Malnutrition and inflammation represent distinct clinical conditions that can mutually influence each other. Certain nutritional deficiencies can compromise mechanisms involving neutrophils, lymphocytes, the complement system, and mucosal immunity—critical components of the body's defense system. This compromise may expose the body to microbial factors, triggering an increase in inflammation indicators. Conversely, symptoms such as loss of appetite, low oral intake, nausea, vomiting, fever, and fatigue, often accompanying inflammation, can lead to malnutrition in patients (Nájera et al., 2004; Scrimshaw & SanGiovanni, 1997).

Recent studies have indicated that the neutrophil‐to‐lymphocyte ratio, an inflammation parameter, serves as a significant marker of patients' nutritional status (Kaya et al., 2019; Wang et al., 2022). This aligns with the findings of the present study, which demonstrated that the CRP‐derived CAR is indicative of the nutritional status of geriatric individuals. The observed outcomes strongly suggest a relationship between inflammation and nutritional status.

Albumin has long been used as a significant indicator of the nutritional status of the individuals (Cabrerizo et al., 2015; Cederholm et al., 2017). The CAR, representing the ratio of CRP to serum albumin, is a novel parameter with the added benefit of being cost‐effective and easily accessible. It incurs no additional costs, is readily calculable, and has demonstrated significant associations with prognosis and specific clinical conditions in various diseases (Liao et al., 2021; Luan et al., 2021; Wu et al., 2022; Yu et al., 2021). Multiple meta‐analyses involving participants with head and neck, colorectal, and urological malignancies have emphasized the CAR as a noteworthy prognostic marker (Liao et al., 2021; Luan et al., 2021; Wu et al., 2022). While numerous studies have explored the relationship between CRP, albumin, and nutritional status, with some mentioned previously (Bellanti et al., 2020; Cederholm et al., 2017; Eckart et al., 2020; Scrimshaw & SanGiovanni, 1997), research specifically focused on the connection between the CAR and nutritional status is limited. A recent study investigating this relationship in 393 inpatients with chronic obstructive pulmonary disease (COPD) identified a CAR value of >3.26 as a significant marker of nutritional status (Baldemir et al., 2022).

To the best of our knowledge, this current study is the first to evaluate the role of the CAR in the nutritional screening of geriatric patients. It determined that an optimal CAR cut‐off value of ≥0.86 predicted nutritional status with a sensitivity of 48.4% and a specificity of 71.7% in geriatric patients. Notably, this CAR cut‐off value is lower than that reported in patients with COPD (Baldemir et al., 2022). It is important to highlight that the present study excluded patients with active inflammatory diseases and CRP values exceeding 10 mg/L, which differs from the other study where 21.9% of patients had pneumonia (Baldemir et al., 2022). This discrepancy may contribute to the variations in CAR cut‐off values between the studies.

The present research is subject to certain limitations. First, the data were collected from a single center, and the study population exclusively consisted of geriatric patients visiting the internal medicine outpatient clinic. Future studies should aim to include a more diverse sample from multiple centers, encompassing individuals of different age groups, including inpatients and residents of nursing homes. This broader approach would enhance the generalizability of the findings. Second, the current study was designed as a cross‐sectional investigation. Future prospective studies, specifically those comparing changes in the CAR with nutritional support interventions, could provide valuable insights. Although CAR was found to be a significant marker of nutritional status, the *r*: −0.196 and area under the curve (AUC): 0.619 on the ROC curve indicate that the results are not very strong.

In conclusion, our findings indicate a significant elevation in the CAR among geriatric patients attending the internal medicine outpatient clinic who were either malnourished or at risk of malnutrition, in comparison to those with normal nutritional status. The CAR exhibited a notable correlation with the nutritional status of these patients and demonstrated predictive capability for identifying geriatric individuals at risk of malnutrition. As a cost‐effective and easily calculable marker, the CAR emerges as a potential independent predictor of malnourishment or the risk of malnutrition in the aging population. This study suggests that the CAR may serve as a valuable tool for nutritional screening in geriatric patients, emphasizing its potential application in clinical practice.

## AUTHOR CONTRIBUTIONS

**Tezcan Kaya**: Conceptualization; methodology; writing—original draft; formal analysis. **Sena Boncuk Ulaş**: Software; writing—review and editing; data curation. **Ahmet Nalbant**: Visualization; project administration. **İlhan Yıldırım**: Software; investigation; data curation. **Kubilay İşsever**: Supervision; validation; project administration. **Cengiz Karacaer**: Visualization; formal analysis. **Cahit Bilgin**: Data curation; writing—original draft. **Asli Vatan**: Validation; methodology. **Türkan Acar**: Project administration; writing—review and editing. **Bilgehan Atılgan Acar**: Writing—review and editing; project administration; supervision. **Yeşim Güzey Aras**: Conceptualization; methodology; visualization. **Mehmet Köroğlu**: Data curation; investigation.

## CONFLICT OF INTEREST STATEMENT

The authors declare no conflicts of interest.

### PEER REVIEW

The peer review history for this article is available at https://publons.com/publon/10.1002/brb3.70017.

## Data Availability

Due to the nature of the research and ethical reasons, supporting data are not available.
